# The Optimal Processing Parameters of Radial Ultrasonic Rolling Electrochemical Micromachining—RSM Approach

**DOI:** 10.3390/mi11111002

**Published:** 2020-11-13

**Authors:** Kailei He, Xia Chen, Minghuan Wang

**Affiliations:** Key Laboratory of Special Purpose Equipment and Advanced Processing Technology, Ministry of Education & Zhejiang Province, Zhejiang University of Technology, Hangzhou 310014, China; hekailei@zjut.edu.cn (K.H.); cx33150@163.com (X.C.)

**Keywords:** microstructure, radial ultrasonic rolling electrochemical micromachining (RUR-EMM), material removal amount, surface roughness, response surface methodology (RSM)

## Abstract

Radial ultrasonic rolling electrochemical micromachining (RUR-EMM) is a new method of electrochemical machining (ECM). By feeding small and rotating electrodes aided by ultrasonic rolling, an array of pits can be manufactured, which is called microstructures. However, there still exists the problem of choosing the optimal machining parameters to realize the workpiece machining with high quality and high efficiency. In the present study, response surface methodology (RSM) was proposed to optimize the machining parameters. Firstly, the performance criteria of the RUR-EMM are measured through investigating the effect of working parameters, such as applied voltage, electrode rotation speed, pulse frequency and interelectrode gap (IEG), on material removal amount (MRA) and surface roughness (*R*_a_). Then, the experimental results are statistically analyzed and modeled through RSM. The regression model adequacies are checked using the analysis of variance. Furthermore, the optimal combination of these parameters has been evaluated and verified by experiment to maximize MRA and minimize *R*_a_. The results show that each parameter has a similar and non-linear influence on the MRA and *R*_a_. Specifically, with the increase of each parameter, MRA increases first and decreases when the parameters reach a certain value. On the contrary, *R*_a_ decreases first and then increases. Under the combined effect of these parameters, the productivity is improved. The experimental value of MRA and *R*_a_ is 0.06006 mm^2^ and 51.1 nm, which were 0.8% and 2.4% different from the predicted values.

## 1. Introduction

The special properties of microstructures, such as drag reduction and noise reduction [1,2], material surface self-cleaning [3], self-repairing [4], antifriction [5], antifatigue [6] and improving load bearing [7], are expected to be widely used in engineering fields such as agricultural machinery, aerospace, mechanical engineering and so on. Generally, the processing material is the metal material that is difficult to process, and for its higher requirements about size, precision, surface quality and so on, result in its higher processing technology requirements. Compared with electrical discharge machining (EDM), laser machining, mechanical machining, etc. [8,9,10], electrochemical machining (ECM), which is widely used in the machining of metal surface microstructure [11,12,13], has the advantages of no loss of processing electrode, no residual stress on the surface after processing, no thermal influence layer, high machining surface quality, etc. With the deepening of research and the pursuit of high efficiency and precision, scholars try to add ultrasonic filed into ECM to explore the effect of multiphysical field machining. Ruszaj et al. [14] confirmed that the surface quality of the workpiece using ultrasonic electrochemical machining is better than pulsed electrochemical machining, and the addition of abrasive powder have a further improvement on the surface quality. Natsu et al. [15] verified that complex vibrations have a more advantageous effect on the replicating accuracy and processing speed than the longitudinal or lateral vibrations individually do in ECM.

The radial ultrasonic rolling electrochemical micromachining (RUR-EMM) combined rolling electrochemical micromachining (R-EMM) and ultrasonic vibration was studied [16], its machining efficiency and the quality of surface machined are both improved with the combination of their benefits. However, affected by many factors, such as applied voltage, pulse frequency, electrode rotation velocity, and the interelectrode gap, microstructure forming size is relatively difficult to control. Besides, due to the effect of ultrasonic, the interaction of each parameter is more complex. Therefore, it is necessary to find a suitable optimization algorithm and establish a theoretical model of the relationship between machining parameters and target parameters to find the best machining parameters.

Many scholars have explored the optimization of ECM. Based on analyzing the effect of parameters including workpiece, electrolyte and cathode, Shang et al. [17] developed a forward feed forward back propagation (BP) neural network to predict the anode accuracy in ECM. Combined BP neural network together with the Levenberg–Marquardt (L–M) optical algorithm, Zhu [18] proposed a digital cathode modification model to accurately design cathode and modify the turbine blade. Xu et al. [19] developed an electrochemical mechanical polishing (ECMP) prediction model on the basis of least squares support vector machines (SVMs) with the radial basis function and assessed the effect of polishing parameters including rotating speed, pressure of grinding tool, current density, grit size and machining time on surface roughness. Using response surface methodology (RSM), Munda et al. [20] established a model between predominant micromachining criteria, i.e., the material removal rate and the radial overcut and electrochemical processing parameters containing machining voltage pulse on/off ratio, machining voltage, electrolyte concentration, voltage frequency and tool vibration frequency and verified the accuracy of the model by ANOVA. Through RSM, Sen et al. [21] set up a significant model between important process parameters of electro jet drilling (EJD) such as applied voltage, capillary outside diameter, feed rate, electrolyte concentration and inlet electrolyte pressure and the quality of hole like roundness error, *R*_a_ and material removal rate (MRR) to improve the hole quality. Using RSM to set the cutting parameters such as the electrolyte concentration, electrolyte flowrate, applied voltage and tool feed rate as the variable and set material removal rate and surface roughness as the response, Senthilkumar et al. [22] optimize the ECM process parameters to maximize MRR and minimize *R*_a_. Ei-Taweel et al. [23] developed a mathematic model to study the performance criteria of the electrochemical turning process through investigating the effect of working parameters, namely, applied voltage, wire feed rate, wire diameter, work piece rotational speed and overlap distance, on the metal removal rate, surface roughness and roundness error.

Based on the above research work, it is clear that the BP neural network, SVM and RSM are both optimal algorithm about the multiparameter. Notably, RSM could match the multiparameter to the multiresponse to analyze the relationship between variable and response. For RUR-EMM, it is necessary to explore the relationship of machining parameters, such as applied voltage, pulse frequency, electrode rotation velocity and interelectrode gap, and target parameters, such as material removal amount and surface roughness to get an optimal parameter, which is a multivariable and multiresponse problem. Therefore, response surface methodology is more applicable to RUR-EMM. In this study, an experiment using response surface methodology with four factors and five levels was designed to analyze and obtain the optimal machining parameters of RUR-EMM. The adequacy of the developed theoretical models was also tested by an analysis of variance (ANOVA) test.

## 2. Experiment Details

### 2.1. Experimental Setup

**Figure 1:**
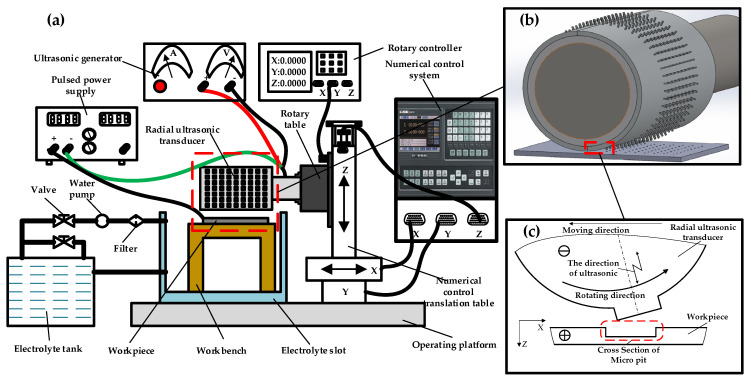
Schematic of (**a**) the proposed radial ultrasonic rolling electrochemical micromachining (RUR-EMM) system; (**b**) machining process and (**c**) machining principle.

The proposed RUR-EMM system shown in Figure 1a comprises major subsystems: ultrasonic generator, pulsed power supply and rotary table controller, the electrolyte slot and rotary table, electrolyte supply, radial ultrasonic transducer and a numerical control laboratory mechanism with three, X, Y and Z, axes motions. As it shown in Figure 1b,c, the workpiece (anode) was fixed on the operating platform when it was processing, and the radial ultrasonic transducer with array micro bulge on the surface, which was clamped on the spindle of the machine tool, was used as the cathode. Under the control of numerical control system and rotation controller, the radial ultrasonic transducer rotated while feeding during machining and converted the electrical signal of ultrasonic generator into ultrasonic vibration. The electrolyte acted as a conductive medium and entered the electrolytic cell through the water pump, filling the entire interelectrode gap. When the pulse power is switched on, pits will be formed on the surface of the work piece due to electrochemical corrosion.

### 2.2. Measurements Procedure

The material removal amount (MRA) of the sample is determined by the size of the cross-sectional area, which was measured using a 3D digital microscope (Keyence, Tokyo, Japan, VHX-7000). Additionally, then the area was calculated by establishing the coordinate and integrating the contour curve. The MRA was specified using the following equation:

(1) $$MRA=S_{section}$$

The *R*_a_ of pit bottom for each specimen was measured by a white light interferometer (Chotest, Shenzheng, China, SuperView W1). 3D image was synthesized by the optical method, the bottom contour of the pit was extracted, and its roughness was measured. Each MRA and *R*_a_’s data was obtained by averaging three measurements. Based on these data, the following RSM optimization analysis was carried out.

## 3. Design of the Response Surface Test

### 3.1. Mathematical Model of Response Surface Methodology

The RSM approach is the procedure for determining the relationship between various process parameters with the various machining criteria and exploring the effect of these process parameters on the coupled responses, i.e., the MRA and *R*_a_. Assuming that y is related to $x_{1},x_{2},\cdots ,x_{p}$, set

(2) $$Ey=f\left(x_{1},\cdots ,x_{p}\right)$$

where $E_{y}$ is the response and $x_{1},\cdots ,x_{p}$ are the coded level of p quantitative variables. For the Equation (1) is unknown, so it is necessary to test (or sample), estimate Equation (1) from the test data obtained from a finite number of tests and use McLaughlin or Taylor expansion formula to fit, namely

(3) $$f\left(x\right)\approx f\left(0\right)+\frac{f^{\prime }\left(0\right)}{1!}x+\frac{f^{\prime \prime }\left(0\right)}{2!}x^{2}+$$

Substitute Equation (2) into Equation (1)

(4) $$Ey=f\left(x_{1},\cdots ,x_{p}\right)\approx a+bx_{1}+\cdots +cx_{p}+\cdots +dx_{p}^{2}+\cdots +ex_{p}^{2}+fx_{1}x_{2}+\cdots +gx_{p-1}x_{p}$$

In order to study the effect of ECM parameters on the two above-mentioned criteria, a second order polynomial response can be fitted into the following equation

(5) $$Ey_{u}=a_{0}+\overset{p}{\underset{i=1}{\sum }}a_{i}x_{i}+\overset{p}{\underset{i=1}{\sum }}a_{ii}x_{i}^{2}+\overset{p}{\underset{i=1}{\sum }}a_{ij}x_{i}x_{j}$$

### 3.2. Experimental Design of Response Surface Machining

Response surface methodology is a collection of mathematical and statistical techniques useful for modeling and optimizing the response variable models involving quantitative independent variables. In order to achieve the accuracy and effectiveness of the experimental program, experiments were carried out according to a central composite design (CCD) based on RSM in this study [24,25].

Applied voltage, electrode rotation speed, pulse frequency and interelectrode gap are important factors affecting the quality of RUR-EMM. Based on the above factors, considering the actual processing conditions in Table 1. The experiments were designed at five levels as shown in Table 2. Table 3 presents the experimental design matrix and experimental results.

### 3.3. Analysis of Response Surface Experimental Results

Using the collected results shown in Table 3, the final regression models for MRA and surface *R*_a_ as determined by the preceding analysis are

(6) $$\begin{array}{ll} R_{MRA}= & -0.14+0.020U+0.15v+4.560\times 10^{-3}f+1.293\times 10^{-3}d\\ & +4.438\times 10^{-4}U\cdot v-1.203\times 10^{-5}U\cdot f\\ & +2.969\times 10^{-6}U\cdot d-1.312\times 10^{-4}v\cdot f+6.875\times 10^{-5}v\cdot d\\ & -1.656\times 10^{-6}f\cdot d-7.003\times 10^{-4}U^{2}\\ & -0.52v^{2}-3.748\times 10^{-4}f^{2}-1.121\times 10^{-5}d^{2} \end{array}$$

(7) $$\begin{array}{ll} R_{Ra}= & 614.12-50.36U-595.90v-14.97f-4.91d+2.30U\cdot v-0.35U\cdot f+0.88U\cdot d+17.76v\cdot f\\ & +1.24v\cdot d-9.081\times 10^{-3}f\cdot d+1.74U^{2}+1324.44v^{2}+1.63f^{2}+0.031d^{2} \end{array}$$

The adequacy of the provided models was checked by the analysis of variance (as Table 4 and Table 5). The significant and non-significant factors were tested through a Student’s *t* test. Design Expert software (version 8.0.6, State Ease Inc., Minneapolis, MN, USA) was used to analyze the experimental data of the response parameters.

## 4. Results and Discussion

### 4.1. Effect of Machining Parameters on the Material Removal Amount

**Figure 2:**
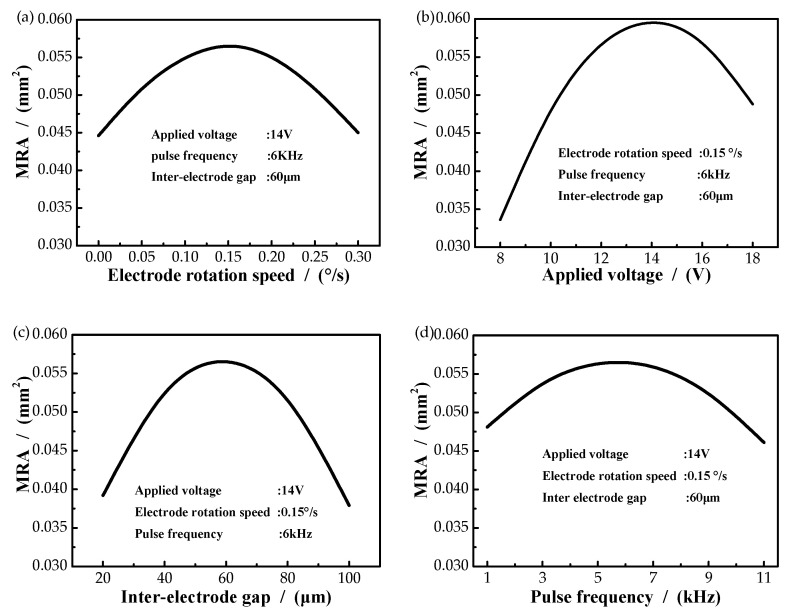
Main effects of process parameters on MRA. (**a**) Electrode rotation speed; (**b**) Applied voltage; (**c**)Interelectrode gap; (**d**)Pulse frequency.

The effect of electrode rotation speed on the MRA is demonstrated in Figure 2a. Obviously, the MRA increased first and then decreased with the increase of the electrode rotation speed. The MRA reached a maximum of 0.0575 mm^2^ when the electrode rotation speed was 0.15°/s. The reason may be that when the applied voltage was stable and the rotating speed was low (less than 0.15°/s), the material exchange in the gap flow field was slow, and this suppressed the material removal on the workpiece surface. However, with the rotating speed increasing, the material exchange in the gap flow field became faster, the electrical conductivity increased in a certain extent, the current density increased and the material removal amount became larger. When the rotational speed was faster than 0.15°/s, the machining time for the workpiece surface deceased and less material will be eroded. Figure 2b shows the effect of applied voltage on MRA. It is obvious that the applied voltage influenced the MRA nonlinearly. Notably, the MRA increased with the increase of the applied voltage until the applied voltage reached a certain value of 14 V, meanwhile, the MRA reached its peak of 0.0596 mm^2^. Then, it decreased. This may be due to the change of electric field. Under the condition of stable flow field, the gap material exchange in electrochemical machining was relatively stable (the increase rate of byproducts in anodic dissolution field was less than that of fluid), resulting in relatively constant conductivity. When the applied voltage was relatively low (less than 14 V), with the increase of the voltage, the gap electric field intensity increased, and with the constant conductivity, the gap current density also increased, leading to a corresponding increase in the material removal amount. When the processing voltage was greater than 14 V, the product increase rate of anodic dissolution field was greater than that of the fluid, and the gap concentration polarization increased, hindering the normal anodic dissolution, resulting in the reduction of the material removal amount.

The effect of the interelectrode gap (IEG) on MRA is shown in Figure 2c. It is noted that the MRA increased with the increase of IEG until it got its peak of 0.555 mm^2^, and then it decreased. This is because under the condition of stable applied voltage, when the IEG was small (less than 60 um), the electric field intensity between electrodes gradually decreased with the increase of the IEG. However, the increased gap made the material exchange of the gap flow field faster and the gap concentration polarization smaller, which was conducive to the dissolution of anode products and led to the increase of material removal amount. However, when the IEG was greater than 60 μm, the material exchange in the clearance flow field was relatively stable and the electrical conductivity was relatively stable. The increased clearance reduced the current density and led to a corresponding decrease in the material removal amount. Figure 2d revealed the influence of pulse frequency of the MRA. It is noted that the MRA increased with the increase of pulse frequency until it got its peak of 0.552 mm^2^, and then it decreased. The reason may be that when the applied voltage is stable and the pulse frequency is low (below 6 kHz), with the increase of pulse frequency, the anodic dissolution time becomes shorter, which is conducive to the material exchange in the gap flow field, leading to the increase of electrical conductivity, current density and material removal amount. However, when the pulse frequency is larger than 6 kHz, the anodic dissolution time becomes less and less, so that the material removal amount decreases.

**Figure 3:**
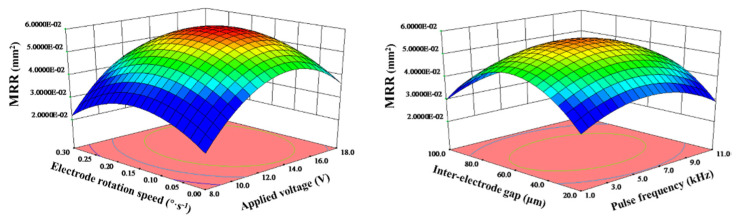
The interactions between process parameters on MRA.

To sum up, each parameter will have an impact on MRA. When applied voltage, electrode rotation speed, pulse frequency and interelectrode gap changed, the value of MRA always increased first and then decreased. Among them, applied voltage had the greatest influence on MRA. When applied voltage changed, the change value of MRA reached 0.0275 mm^2^. Figure 3 shows the interactions between process parameters on MRA. The curved surface of MRA was like a ‘convex hull’, there was a maximum at the peak point under the interaction of parameters.

### 4.2. Effect of Machining Parameters on Surface Roughness

**Figure 4:**
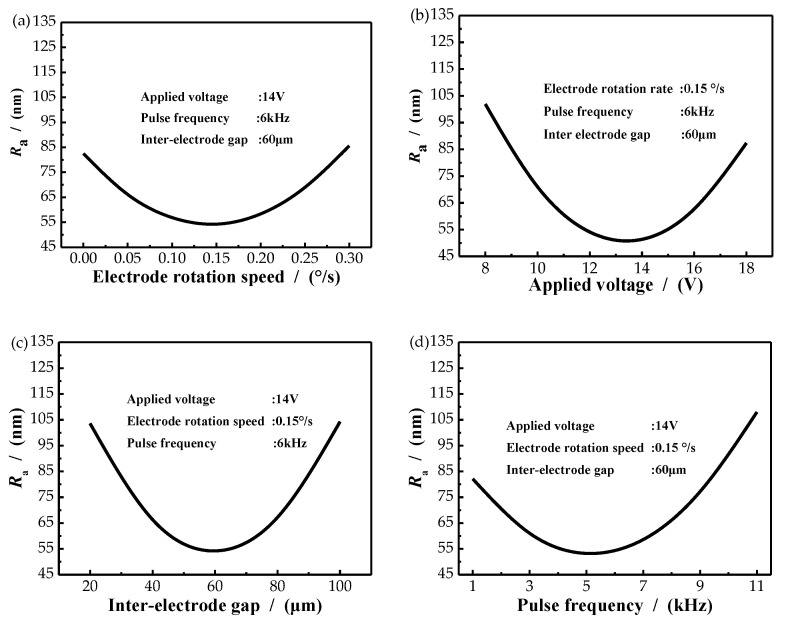
Main effects of process parameters on *R*_a._ (**a**)Electrode rotation speed; (**b**) Applied voltage; (**c**) Interelectrode gap; (**d**) Pulse frequency.

Surface roughness (*R*_a_) can display the quality of a machined surface. It is necessary to investigate the effects of parameters on *R*_a_. Figure 4a shows the influence of electrode rotation speed on *R*_a_. *R*_a_ decreased from 83 to 55 nm when the electrode rotation speed increased from 0 to 0.15°/s, and then it increased from 55 to about 84 nm when electrode rotation speed increased from 0.15 to 0.5°/s. This is because under the condition of stable applied voltage, at a low speed (less than 0.15°/s), with the increase of speed, the material exchange in the gap flow field became faster, the conductivity increased to a certain extent, the current density increased and the surface roughness decreased. When the rotational speed was faster than 0.15°/s, the surface processing time of the material was reduced to less anodic dissolution. Coupled with rotation, the electric field between poles was not uniform, resulting in worse surface roughness. The effect of applied voltage on *R*_a_ is shown in Figure 4b. *R*_a_ decreased from 104 to 47 nm when applied voltage increased from 8 to 14 V, and then it increased from 47 to about 90 nm when applied voltage increased from 14 to 16 V. A reason may be given that under the condition of a stable flow field, the gap material exchange in electrochemical machining was relatively stable (the increase rate of products in anodic dissolution field was less than that of fluid), resulting in relatively constant gap conductivity. When the applied voltage was relatively low (less than 14 V), with the increase of the voltage, the gap electric field intensity increased, and with the constant conductivity, the gap current density also increased, leading to a corresponding increase in the material removal amount. When the processing voltage was greater than 14 V, the product of anodic dissolution field increased faster than the fluid velocity, and the gap electric field was not evenly distributed, resulting in the decrease of surface roughness.

The effect of the interelectrode gap (IEG) is illustrated in Figure 4c. *R*_a_ decreased from 104 to 50 nm when IEG increased from 40 to 60 μm, and then it increased from 50 to about 106 nm when IEG increased from 60 to 80 μm. This result can be explained that under the condition of stable applied voltage, when the processing gap was small (less than 60 um), the electric field intensity between electrodes gradually decreased with the increase of the IEG. However, the increased clearance makes the material exchange of the clearance flow field faster and the gap concentration polarization smaller, which was conducive to the dissolution of anode products and led to the increase of surface roughness. However, when the machining gap was greater than 60 μm, the material exchange in the gap flow field was relatively stable and the electrical conductivity was relatively stable. The increased IEG reduced the current density and led to a corresponding decrease in surface roughness. Figure 4d revealed the effect of pulse frequency on *R*_a_. *R*_a_ decreased from 84 to 47 nm when pulse frequency increased from 2 to 6 kHz, and then it increased from 47 to about 110 nm when pulse frequency increased from 60 to 80 μm. It can be explained that when the applied voltage was stable and the pulse frequency was low (less than 6 kHz), with the increase of the pulse frequency, the anodic dissolution time became shorter, which is conducive to the material exchange of the clearance flow field, the uniform distribution of the electric field between poles, and the decrease of the surface roughness. However, when the pulse frequency was larger than 6 kHz, the anodic dissolution time became less and less, so that the surface roughness increased.

**Figure 5:**
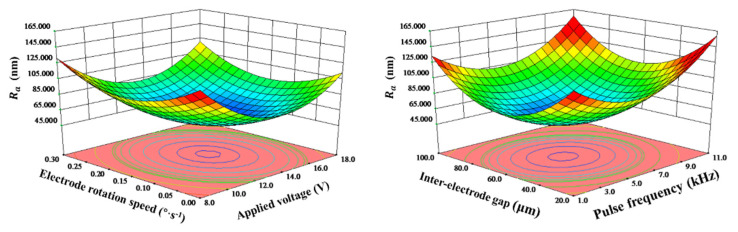
The interactions between process parameters on *R*_a_.

Above all, each parameter will have an impact on *R*_a_. When applied voltage, electrode rotation speed, pulse frequency and interelectrode gap varied, the value of *R*_a_ always decreased first and then increased. Among them, applied voltage had the greatest influence on *R*_a_. When applied voltage changed, the change value of MRA reached 57 nm. Figure 5 shows the interactions between process parameters on *R*_a_. The curved surface of *R*_a_ was like a ‘valley’, *R*_a_ would reach its minimum at the bottom point under the interaction of parameters.

### 4.3. Multiresponse Optimization of the Process

Selection of the optimal machining parameter combination for achieving improved process performance, e.g., material removal amount and surface roughness, is a challenging task in RUR-EMM due to the presence of a large number of process variables and complicated stochastic process mechanisms. Based on the experimental results data shown in Section 4.1 and Section 4.2, it could be seen there existed the optimal parameters for the maximum MRA and the minimum *R*_a_ of the machined workpiece surface.

**Figure 6:**
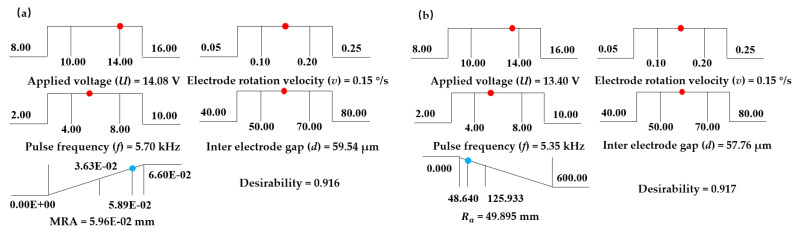
Optimal machining parameters with (**a**) maximum MRA and (**b**) minimum *R*_a_.

**Figure 7:**
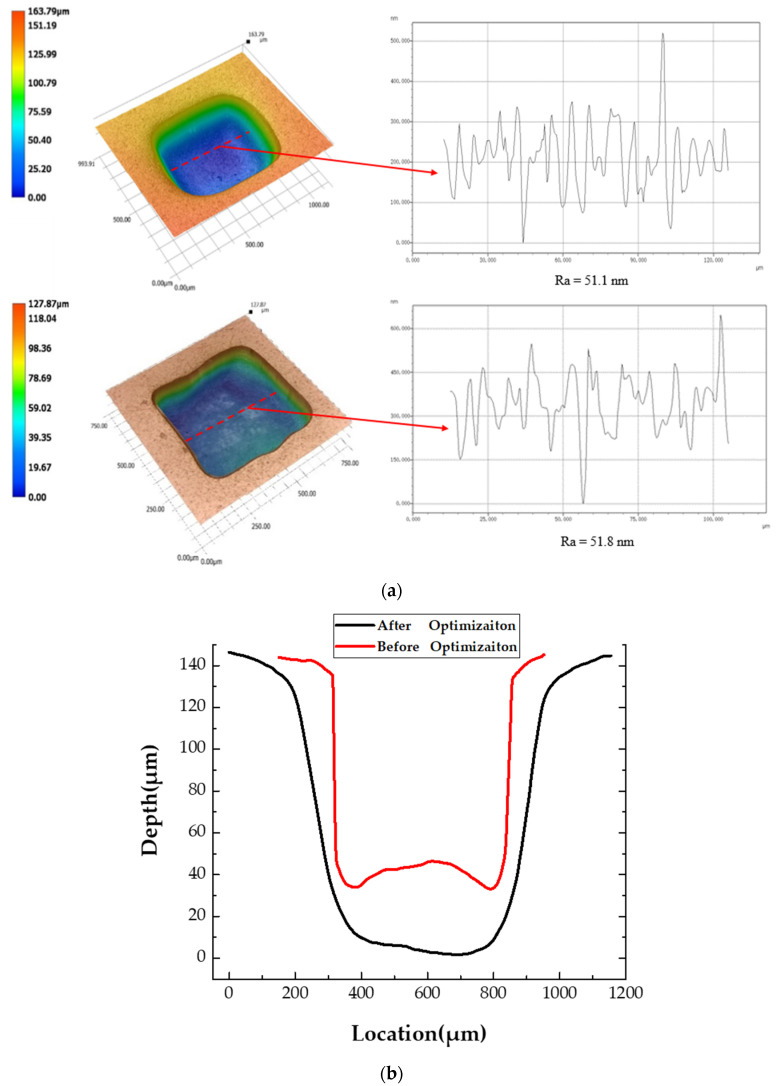
The *R*_a_ and MRA before and after optimization. (**a**) The morphology of micro pit and its *R*_a_; (**b**) The cross section of micro pit.

At the same time, the distribution diagram of the optimal machining parameters for MRA and *R*_a_ was analyzed. Figure 6 shows the distribution of optimal machining parameters with maximum MRA, where applied voltage (*U*) was 14.08 V, electrode rotation velocity (*v*) was 0.15°/s, pulse frequency(*f*) was 5.70 kHz, interelectrode gap (*d*) was 59.54 µm and corresponding MRA was 0.0596 mm^2^. Figure 7 shows the distribution of optimal machining parameters with the minimum *R*_a_, where applied voltage (*U*) was 13.40 V, electrode rotation velocity (*v*) was 0.15°/s, pulse frequency (*f*) was 5.35 kHz, interelectrode gap (*d*) was 57.76 µm and the corresponding *R*_a_ was 49.895 nm. Therefore, the average value from each parameter was approximately seen as an optimal parameter shown in Table 6.

A verification experiment was performed using the optimized parameters shown in Table 4. After the experiment of the optimal processing parameters, the MRA of the micro dimple was 0.06006 mm^2^ and *R*_a_ was 51.1 nm, which were 0.8% and 2.4% different from the predicted values, respectively. Figure 7 shows the cross sectional area of the micro pit and its *R*_a_. Clearly, the value of MRA was greater than the maximum MRA (0.05649 mm^2^) without optimization, and the value of *R*_a_ was less than the minimum *R*_a_ (51.8 nm) without optimization, indicating that the optimization model was effective and successful.

## 5. Conclusions

Based on the experimental method of the Central Composite Design, 31 experimental studies using radial ultrasonic rolling electrochemical micromachining (RUR-EMM) under different working conditions were carried out, and according to the response surface optimization analysis, global optimization was carried out by using the Design Expert software, and the combination of optimization parameters in a certain range was found. The following conclusion can be drawn:

(1) Response surface methodology is a suitable data optimization algorithm to radial ultrasonic rolling electrochemical micromachining.
(2) Parameters, including applied voltage, electrode rotation speed, pulse frequency and the interelectrode gap all had a nonlinear effect on the MRA and *R*_a_. Especially, the applied voltage has.
(3) The optimum combination of parameters of applied voltage 14.70 V, electrode rotation speed 0.15°/s, pulse frequency 5.5 kHz, interelectrode gap 58.6 µm for maximizing the metal removal rate of 0.06006 mm^2^ and a minimizing surface roughness of 51.1 nm could be obtained.

## Figures and Tables

**Table 1:** Processing conditions.

Parameter	Value
Electrolyte concentration	10%NaNO_3_
Electrolyte temperature (*T_e_*)	25°
Protrusion size	70 mm × 50 mm
Ultrasonic vibration frequency(*f_u_*)	28 KHz
Amplitude (A)	10 μm
Thickness of workpiece (*T_w_*)	0.3 mm
Processing time	5 min
Power supply duty cycle	0.5
Workpiece material	SS 304

**Table 2:** Machining parameters and their levels.

Parameters	Units	Level				
		−2	−1	0	+1	+2
Applied voltage (*U*)	V	8	10	12	14	16
Electrode rotation speed (*v*)	°/s	0.05	0.15	0.1	0.2	0.25
Pulse frequency (*f*)	kHz	2	4	6	8	10
Inter-electrode gap (*d*)	μm	40	50	60	70	80

**Table 3:** Experimental results.

Run	Factors				Responses		Run	Factors					Responses
	*U* V	*v* °/s	*f* kHz	*d* μm	MRA mm^2^	$R_{a}$ of Pit Bottom nm		*U* V	*v* °/s	*f* kHz	*d* μm	MRA mm^2^	$R_{a}$ of Pit Bottom nm
1	8	0.05	4	80	0.03025	116.7	17	12	0.15	6	80	0.05425	110.0
2	8	0.15	6	60	0.03634	179.5	18	12	0.15	10	60	0.05217	135.0
3	8	0.25	2	40	0.02826	200.5	19	12	0.25	6	60	0.05381	136.9
4	8	0.25	2	80	0.02814	209.9	20	14	0.1	4	50	0.05510	51.8
5	10	0.1	4	50	0.04321	112.9	21	14	0.1	4	70	0.05479	56.9
6	10	0.1	4	70	0.04290	116.6	22	14	0.1	8	50	0.05408	70.7
7	10	0.1	8	50	0.04291	130.4	23	14	0.1	8	70	0.05381	80.3
8	10	0.1	8	70	0.04173	139.3	24	14	0.2	4	50	0.05533	73.2
9	10	0.2	4	50	0.04337	133.7	25	14	0.2	4	70	0.05490	82.0
10	10	0.2	4	70	0.04302	140.7	26	14	0.2	8	50	0.05432	98.2
11	10	0.2	8	50	0.04241	160.0	27	14	0.2	8	70	0.05399	104.0
12	10	0.2	8	70	0.04212	171.9	28	16	0.05	10	80	0.03981	139.0
13	12	0.05	6	60	0.05337	102.1	29	16	0.15	6	60	0.04889	115.2
14	12	0.15	2	60	0.05345	105.6	30	16	0.25	2	80	0.04058	146.0
15	12	0.15	6	40	0.05439	109.7	31	16	0.25	10	40	0.03972	175.0
16	12	0.15	6	60	0.05649	107.6	-	-	-	-	-	-	-

**Table 4:** Analysis of variance for the material removal amount (MRA).

Source	Sum of Squares	DF	Mean Square	F Value	Prob > F	
Model	1.121 ×10^−3^	1	8.004 × 10^−5^	19.68	<0.0001	significant
*U*	1.808 ×10^−4^	1	1.808 × 10^−4^	44.45	<0.0001	-
*v*	8.736 ×10^−6^	1	8.736 × 10^−6^	2.15	0.1622	-
*f*	1.307 ×10^−5^	1	1.307 × 10^−5^	3.21	0.0920	-
*d*	1.977 ×10^−5^	1	1.977 × 10^−5^	4.86	0.0425	-
*U*·*v*	3.151 ×10^−8^	1	3.151 × 10^−8^	7.745 × 10^−3^	0.9310	-
*U*·*f*	3.706 ×10^−8^	1	3.706 × 10^−8^	9.109 × 10^−3^	0.9251	-
*U*·*d*	5.641 × 10^−8^	1	5.641 × 10^−8^	0.014	0.9077	-
*v*·*f*	2.756 × 10^−9^	1	2.756 × 10^−9^	6.775 × 10^−4^	0.9796	-
*v*·*d*	1.891 × 10^−8^	1	1.891 × 10^−8^	4.647 × 10^−4^	0.9465	-
*f*·*d*	1.756 × 10^−8^	1	1.756 × 10^−8^	4.316 × 10^−3^	0.9484	-
*U* ^2^	2.244 × 10^−4^	1	2.244 × 10^−4^	55.16	<0.0001	-
*v* ^2^	4.855 × 10^−5^	1	4.855 × 10^−5^	11.93	0.0033	-
*f* ^2^	6.426 × 10^−5^	1	6.426 × 10^−5^	15.80	0.0011	-
*d* ^2^	3.593 × 10^−5^	1	3.593 × 10^−6^	8.83	0.0090	-
Residual	6.509 × 10^−5^	16	4.068 × 10^−6^	-	-	-
Lack of fit	6.509 × 10^−5^	10	6.509 × 10^−6^	-	-	-
Pure Error	0.000	6	0.000	-	-	-
Cor Total	1.186 × 10^−3^	30	-	-	-	-

**Table 5:** Analysis of variance for *R*_a_.

Source	Sum of Squares	DF	Mean Square	F Value	Prob > F	
Model	15508	14	1107.72	6.02	0.0005	significant
*U*	1849.65	1	1849.65	10.06	0.0059	-
*v*	203.48	1	203.48	1.11	0.3085	-
*f*	209.42	1	209.42	1.14	0.3017	-
*d*	388.84	1	388.84	2.11	0.1652	-
*U*·*v*	1.90	1	1.90	0.010	0.9203	-
*U*·*f*	48.36	1	48.36	0.26	0.6151	-
*U*·*d*	114.69	1	114.69	0.62	0.4412	-
*v*·*f*	76.15	1	76.15	0.41	0.5290	-
*v*·*d*	13.34	1	13.34	0.073	0.7911	-
*f*·*d*	0.85	1	0.85	4.620 × 10^−3^	0.9467	-
*U* ^2^	1714.33	1	1714.33	9.32	0.0076	-
*v* ^2^	386.14	1	386.14	2.10	0.1666	-
*f* ^2^	1458.79	1	1458.79	7.93	0.0124	-
*d* ^2^	339.94	1	339.94	1.85	0.1928	-
Residual	2942.13	16	183.88	-	-	-
Lack of fit	2942.13	10	204.44	-	-	-
Pure Error	0.000	6	0.000	-	-	-
Cor Total	18450.21	30	-	-	-	-

**Table 6:** Optimum value of parameters.

Parameter	Optimum Value
Applied voltage (V)	14.7
Electrode rotation speed (°/s)	0.15
Pulse frequency (kHz)	5.5
Inter-electrode gap (μm)	58.6
